# Effect of a carbohydrate-rich beverage on rate of cesarean delivery in primigravidae with epidural labor analgesia: a multicenter randomized trial

**DOI:** 10.1186/s12884-022-04659-2

**Published:** 2022-04-19

**Authors:** Ting Ding, Chun-Mei Deng, Xiao-Feng Shen, Yao-Wu Bai, Xiao-Lan Zhang, Ji-Ping Liu, Li-Juan Yang, Hai-Tao Yu, Lei Xie, Hong Chen, Dong-Liang Mu, Yuan Qu, Hui-Xia Yang, Ai-Rong Bao, Sai-Nan Zhu, Dong-Xin Wang

**Affiliations:** 1 Department of Anesthesiology and Critical Care Medicine, Peking University First Hospital, No.8 Xishiku street, Beijing, 100034 China; 2 Department of Anesthesiology, Woman’s Hospital of Nanjing Medical University, Nanjing, Jiangsu China; 3 Department of Anesthesiology, Tangshan Maternity and Child Health Care Hospital, Tangshan, Hebei China; 4 Department of Anesthesiology, Gansu Provincial Maternity and Child Care Hospital, Lanzhou, Gansu China; 5 Department of Anesthesiology, Foshan Maternal and Child Health Hospital, Foshan, Guangdong China; 6 Department of Anesthesiology, Urumqi Women and Child Health Care Hospital, Urumqi, Xinjiang, China; 7 Department of Anesthesiology, Linyi people’s hospital, Linyi, Shandong China; 8 Department of Anesthesiology, Anhui Women and Child Health Care Hospital, Hefei, Anhui China; 9 Department of Anesthesiology, Women’s Hospital of Zhejiang University, Zhejiang, Hangzhou China; 10 Department of Obstetrics and Gynecology, Peking University First Hospital, Beijing, China; 11 Department of Biostatistics, Peking University First Hospital, Beijing, China; 12 Outcomes Research Consortium, Cleveland, OH USA

**Keywords:** Carbohydrate-rich beverage, Epidural labor analgesia, Incidence of cesarean delivery, Neonatal hypoglycemia

## Abstract

**Background:**

Labor represents a period of significant physical activity. Inefficient energy supply may delay labor process and even lead to cesarean delivery. Herein we investigated whether ingestion of a carbohydrate-rich beverage could reduce cesarean delivery in laboring women with epidural analgesia.

**Methods:**

This multicenter randomized trial was conducted in obstetrician-led maternity units of nine tertiary hospitals in China. Primigravidae with single term cephalic pregnancy who were preparing for vaginal birth under epidural analgesia were randomized to intake a carbohydrate-rich beverage or commercially available low-carbohydrate beverages during labor. The primary outcome was the rate of cesarean delivery. Secondary outcomes included maternal feeling of hunger, assessed with an 11-point scale where 0 indicated no hunger and 10 the most severe hunger, and maternal and neonatal blood glucose after childbirth.

**Results:**

Between 17 January 2018 and 20 July 2018, 2008 women were enrolled and randomized, 1953 were included in the intention-to-treat analysis. The rate of cesarean delivery did not differ between the two groups (11.3% [111/982] with carbohydrate-rich beverage vs. 10.9% [106/971] with low-carbohydrate beverages; relative risk 1.04, 95% CI 0.81 to 1.33; *p =* 0.79). Women in the carbohydrate-rich beverage group had lower subjective hunger score (median 3 [interquartile range 2 to 5] vs. 4 [2 to 6]; median difference − 1; 95% CI − 1 to 0; *p <* 0.01); their neonates had less hypoglycemia (1.0% [10/968] vs. 2.3% [22/956]; relative risk 0.45; 95% CI 0.21 to 0.94; *p =* 0.03) when compared with those in the low-carbohydrate beverage group. They also had higher rates of maternal hyperglycemia (6.9% [67/965] vs. 1.9% [18/953]; *p <* 0.01) and neonatal hyperglycemia (9.2% [89/968] vs. 5.8% [55/956]; *p <* 0.01), but none required special treatment.

**Conclusions:**

For laboring primigravidae with epidural analgesia, ingestion of a carbohydrate-rich beverage compared with low-carbohydrate beverages did not reduce cesarean delivery, but relieved maternal hunger and reduced neonatal hypoglycemia at the expense of increased hyperglycemia of both mothers and neonates. Optimal rate of carbohydrate supplementation remains to be determined.

**Trial registration:**

www.chictr.org.cn; identifier: ChiCTR-IOR-17011994; registered on 14 July 2017.

**Supplementary Information:**

The online version contains supplementary material available at 10.1186/s12884-022-04659-2.

## Introduction

Fasting during labor was once the routine practice because of the concerns of aspiration in case of emergent cesarean delivery under general anesthesia [1]. In recent years, this restriction policy begins to relax due to the rarity of pulmonary aspiration as well as the improvement in obstetric anesthesia practice [2]. The American College of Obstetricians and Gynecologists recommends that “oral intake of modest amounts of clear liquids (e.g., water, black coffee, and sports drinks) may be allowed for patients with uncomplicated labor”; but “solid foods should be avoided in laboring patients” [3]. This is congruent with the American Society of Anesthesiologists’ guidelines [4]. The Chinese Society of Anesthesiologists Task Force on Obstetric Anesthesia encourages parturients to ingest an energy-rich, low-residue beverage during epidural labor analgesia [5]. In China, commercial rehydration beverages with low carbohydrate content are generally allowed in parturients with epidural analgesia [6].

Uterine contraction during labor generates laboring pain and stress, and significantly increases the metabolic rate and glucose consumption [7]. At the same time, parturients dehydrate easily and require adequate hydration to ensure efficient childbirth [8]. The energy consumption of parturients in labor is not well documented but may be comparable to a continuous moderate aerobic exercise [9], which is about 50 to 100 kcal/h [10]. In sports medicine, carbohydrate-rich beverage is proved to enhance the capacity of performance and delay the occurrence of fatigue during prolonged moderate-to-high intensity exercise [11]. Oral rehydration solution with carbohydrate polymers has been designed for enhanced recovery after surgery [12]. For example, maltodextrin can provide sufficient glucose to stimulate insulin secretion to restore glycogen stores, similar to the effect of a meal [13]. Carbohydrate-containing drinks have been widely used in peripartum women with restriction to diet [14].

Energy depletion and inadequate hydration during labor may alter the acid-base balance, reduce the strength of uterine myometrium and skeletal muscles, prolong labor, and increase cesarean delivery [8, 15]. Indeed, lack of caloric intake was associated with a higher incidence of instrumental delivery due to non-progressing second stage [16]. However, available evidence showed that oral carbohydrate ingestion has no effect on labor outcomes [14, 17–20]. It is noted that, in those studies, the quantity of caloric intake in carbohydrate drink group was low (14 to 47 kcal/h), which may not meet the energy requirement of women in labor. Furthermore, those early studies compared the effect of carbohydrate supplementation with no oral intake or a carbohydrate-free drink, whereas commercial rehydration beverage with low carbohydrate content has become a common practice. The impact of a carbohydrate-rich beverage on the process of labor remains to be determined.

We therefore performed this multicenter trial to test the hypothesis that, for laboring primigravidae with epidural analgesia, hydration with a carbohydrate-rich beverage compared with commercially available low-carbohydrate beverages may reduce the incidence of cesarean delivery.

## Methods

### Design and setting

This was a multicenter randomized controlled trial with two parallel arms to test a superiority hypothesis. The study protocol (Additional file 1: File S1) was approved by the Clinical Research Ethics Committee of Peking University First Hospital (2017–1360; on 9 June 2017) and other participating centers and registered in Chinese Clinical Trial Registry (www.chictr.org.cn; identifier: ChiCTR-IOR-17011994; on 14 July 2017). Written informed consent was obtained from each participant prior to the recruitment. The trial was conducted in labor and delivery units of nine tertiary hospitals in China (Additional file 2: Table S1). All participating centers were provided with access to a study-specific Research Electronic Data Capture (REDCap) database [21], and were responsible for data entry for their enrolled patients. The study provided a designated pathway for eligible women to promptly get carbohydrate-rich beverage, which was not routinely available to others at the trial centers. This trial is adhered to CONSORT guidelines.

### Study population

Women were screened at delivery room admission. The inclusion criteria were primigravidae with a single cephalic pregnancy at or beyond 37 weeks who were admitted for vaginal birth and requested epidural labor analgesia. The exclusion criteria included the following: age < 18 or > 34 years, comorbid/gestational diabetes mellitus [22], suspected fetal abnormalities by prenatal ultrasonography, presence of contraindications to epidural analgesia (including history of infectious disease of the central nervous system, history of spinal disease, systemic infection, skin or soft tissue infection at the site of puncture, coagulopathy, severe low-back/lower extremity pain, and body mass index > 35 kg/m^2^), or other severe gestational comorbidity such as hemolysis, elevated Liver enzymes, and low platelets (HELLP) syndrome.

### Sample size

In a pilot trial of our own, the rate of cesarean delivery was 8% of primigravidae with carbohydrate-rich drink and 12% of those with low-carbohydrate commercial beverages [23]. We assumed similar rates in the present study. With the significance level set at 0.05 (two-sided) and power set at 80%, the sample size required to detect difference was 1760 participants. Considering a drop-out rate of about 10%, we planned to enroll 2000 participants. Sample size calculation was performed with the Stata 10.0 software (Stata Corp. LP, College Station, TX, USA).

### Randomization and intervention

A biostatistician, who was independent of data management and statistical analyses, generated random numbers in a 1:1 ratio with a block size of 4 using the SAS 9.2 software (SAS Institute, Cary, NC). Randomization was stratified according to the study centers. The results of randomization were sealed in sequentially numbered opaque envelopes and stored at the sites of investigation. During the study period, a trial coordinator was designated in each center and was responsible to open the randomization letter according to the recruitment sequence of each participant shortly before starting epidural labor analgesia. In this way the enrolled participants were randomly assigned into two groups.

After initiating epidural labor analgesia, women in the carbohydrate-rich group were instructed to drink as will a carbohydrate-rich beverage [Outfast®: 285 mOsm/kg, 14.1% carbohydrate (maltodextrin 100 g/L, fructose 23.9 g/L, glucose 16.9 g/L), Na^+^ 2.0 mmol/L, K^+^ 4.9 mmol/L, 0.58 kcal/ml; Yichang Humanwell Pharmaceutical Co, Ltd., Hubei, China]; women in the low-carbohydrate group were instructed to drink as will commercially available low-carbohydrate beverages (Pocarisweat®: 0.26 kcal/ml, Otsuka Pharmaceutical Co. Ltd., Tokyo, Japan; Gatorade®, 0.24 kcal/ml, PepsiCo, Chicago, IL, USA; or Mizone®: 0.21 kcal/ml, Danone, Guangzhou, Guangdong, China). All participants had free access to water, but solid foods were not permitted. Dietary intake prior to labor analgesia and after the third stage of labor was not limited.

Investigators who assessed/collected outcomes including intrapartum cesarean delivery, maternal sensation of hunger and thirst, and maternal and neonatal blood glucose were not involved in intrapartum care and had no knowledge of study group assignment. There was no blindness provided, otherwise, because of the different appearance in study beverages.

### Execution

Epidural labor analgesia was initiated per parturient request after active labor independent to cervical dilation. The ASA standard monitoring applied, and intravenous Ringer’s lactate solution was provided during epidural placement. An epidural catheter was inserted between L2–3 or L3–4 intervertebral space. After confirming the catheter position with loss of resistance and negative aspiration, a 10 ml mixture of 0.1% ropivacaine (AstraZeneca AB, Södertälje, Sweden) and 0.5 μg/ml sufentanil (EuroCept BV, Ankeveen, Netherlands) was administered as a loading dose; an additional 5 ml mixture was administered 10 min later if the Numeric Pain Rating Scale (NPRS, an 11-point scale where 0 = no pain and 10 = the worst pain) remained ≥4. A programmed intermittent epidural bolus pump (ZZB-II; Jiangsu Aipeng Medical Science and Technology Company Ltd., Nantong, China) was attached 30 min later, which was established with 200 ml mixture of 0.07% ropivacaine and 0.45 μg/ml sufentanil, and programmed to deliver 4 ml in 60 min with 6-ml controlled boluses at a 20-min lockout interval. The maximal volume was 26 ml/h. The pump was stopped at the end of the third stage of labor.

During labor, maternal vital signs were routinely monitored every 1 to 2 h and more frequently as necessary. Continuous external fetal heart rate monitoring and/or tocodynamometry were applied as indicated. Obstetric managements such as oxytocin administration and forceps assisted/cesarean delivery were decided by obstetricians; cesarean delivery was performed for indications including cephalopelvic disproportion, dystocia, failure to progress, fetal distress, and intrauterine infection, according to Chinese guidelines [24]. In case of emergent cesarean delivery, epidural anesthesia was converted from the epidural labor analgesia catheter. For parturients requiring general anesthesia for various reasons, a rapid sequence induction with endotracheal intubation under cricoid pressure was performed. Antacid prophylaxis and nasogastric tube were not routinely used.

The rooming-in policy is routinely applied in all participating centers. All newborns were observed for 2 h in the delivery room before being transferred to the postpartum ward. Breastfeeding was encouraged whenever possible.

Adverse events were monitored from the initiation of epidural analgesia to leaving for postpartum ward. Maternal hypoglycemia was defined as blood glucose < 3.3 mmol/L [25] and managed with oral or intravenous 5% glucose. Maternal hyperglycemia was defined as blood glucose > 11.1 mmol/L [26]; insulin was administered prudently when considered necessary. Neonatal hypoglycemia was defined as blood glucose < 2.6 mmol/L [27, 28] and was treated by feeding with 5% glucose solution (10-ml per dose). Neonatal hyperglycemia was defined as blood glucose > 7.0 mmol/L [29]; intervention was not given unless blood glucose > 10 mmol/L [29, 30]. Antiemetics such as 5-HT3 receptor antagonists (tropisetron) and/or glucocorticoids (dexamethasone) were provided during cesarean delivery for prophylaxis of postoperative nausea and vomiting.

### Data collection and outcomes

Baseline data including demographic characteristics, gestation weeks, gravidity, comorbidities, obstetric complications, and antepartum hemoglobin were recorded. Maternal sensation of hunger and thirst was self-evaluated using the Numeric Rating Scale (NRS; an 11-point scale where 0 indicated no hunger/thirst and 10 indicated the most severe hunger/thirst) at the initiation of epidural labor analgesia and before leaving the delivery room. Maternal blood glucose level was tested using a blood glucose monitor (Accu-Chek®, Roche, Germany) at the same time-point.

Intrapartum maternal variables included oxytocin requirements, artificial membrane rupture, dosage of labor analgesia, duration of labor, episiotomy, mode of delivery, maximal temperature, estimated blood loss, volume of oral intake, and volume of intravenous fluid. The NPRS were assessed before analgesia, at 10 and 30 min after analgesia, and at full cervical dilation. Total fluid intake (oral and intravenous) as well as calories supplied by oral and intravenous fluid were calculated. The data of newborns including sex, birth weight [31, 32], Apgar scores at 1 and 5 min after birth, and neonatal intensive unit admission were collected. Neonatal blood glucose level was tested instantly after birth using a blood glucose monitor (Accu-Chek®, Roche, Germany), and was repeated 30 min later when necessary.

Our primary outcome was the rate of cesarean delivery. Secondary outcomes included duration of labor, rate of forceps delivery, subjective hunger/thirst NRS score after childbirth, maternal/neonatal blood glucose, 1- and 5-min Apgar scores, the umbilical artery pH value, and the rate of neonatal ward admission.

### Statistical analysis

The balance of baseline data between groups was assessed using absolute standardized difference, calculated as the absolute difference in means, medians, or proportions divided by the pooled standard deviation [33]. Baseline variables with an absolute standardized difference (ASD) of ≥0.089 (i.e., 1.96× $\sqrt{\left(\mathsf{n}\mathsf{1}+\mathsf{n}\mathsf{2}\right)/\left(\mathsf{n}\mathsf{1}\times \mathsf{n}\mathsf{2}\right)}$) were considered imbalanced and were adjusted for in analyses when necessary.

The primary outcome, i.e., the rate of cesarean delivery, was compared with chi-square test, with difference between groups expressed as relative risk (and 95% CI). Imbalanced baseline and/or intrapartum variables were included in a logistic regression model to adjust for potential confounding effects on the primary outcome. Exploratory analyses were performed to assess differences of the primary outcome in predefined subgroups including study site, age, body mass index, prepartum anemia, comorbidity, and obstetric complications. Treatment-by-covariate interactions were assessed separately for each subgroup factor using logistic regression. Other numeric variables, such as NPRS or NRS, were analyzed with independent sample t-test or Mann-Whitney U test. Median differences (and 95% CI) were calculated with Hodges-Lehmann estimators. Categorical variables were analyzed with chi-square test, continuity-corrected chi-square test, or Fisher’s exact test. Missing data were not replaced.

Analyses were performed in the intention-to-treat population. We also did per-protocol analysis for the primary outcome. Interim analysis was not performed. Two-tailed *p* values of < 0.05 were considered statistically significant. For the treatment-by-covariate interaction in predefined subgroup analyses, *p* values of < 0.10 were considered statistically significant. Statistical analyses were done on SPSS 25.0 software (IBM SPSS, Chicago, IL) and SAS 9.2 software (SAS Institute, Cary, NC).

## Results

### Patient population

**Fig. 1:**
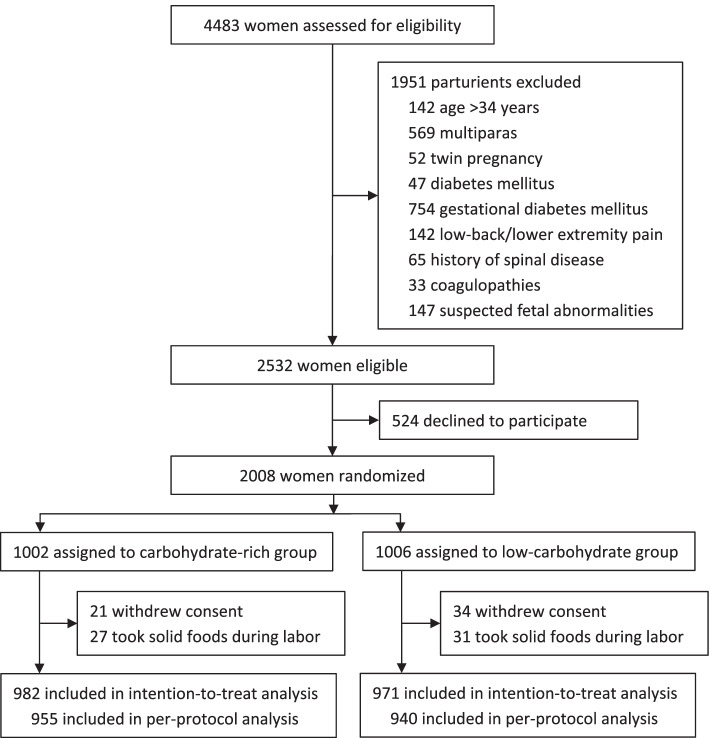
Flow chart of the study

Between 17 January 2018 and 20 July 2018, 2008 parturients were enrolled and randomly assigned to receive either the carbohydrate-rich beverage (carbohydrate-rich group; *n =* 1002) or the commercial low-carbohydrate beverages (low-carbohydrate group; *n =* 1006). During the study period, 55 parturients withdrew consents; 58 parturients violated the protocol and took solid foods during labor. At last, 1953 parturients were included in the intention-to-treat analysis and 1895 parturients were included in the per-protocol analysis (Fig. 1).

### Baseline and intrapartum data

The two groups were well comparative (Table 1). Among intrapartum variables, the volumes of oral carbohydrate beverage and total fluid intake were higher in the carbohydrate-rich group than in the low-carbohydrate group. As expected, the amounts of oral and total calorie intake were higher in the carbohydrate-rich group than in the low-carbohydrate group; total calorie intake per hour was median 65 kcal/h (interquartile range 46 to 88) with carbohydrate-rich beverage vs. 18 kcal/h (7 to 27) with low-carbohydrate beverages (*p* < 0.01; Table 2).

**Table 1:** Baseline characteristics

	Carbohydrate-rich beverage (*n =* 982)	Low-carbohydrate beverages (*n =* 971)	ASD
Age (year)	28.3 ± 3.6	28.3 ± 3.5	0.002
Body Mass Index (kg/m2)	27.0 ± 3.1	27.0 ± 3.0	0.002
Duration of gestation (day)	277 ± 19	276 ± 21	0.013
Gravidity	1 [1, 2]	1 [1, 2]	0.012
Medical comorbidity ^a^	46 (4.7%)	60 (6.2%)	0.071
Gynecological disease ^b^	42 (4.3%)	49 (5.0%)	0.038
Obstetric disease ^c^	26 (2.6%)	37 (3.8%)	0.072
Prepartum hemoglobin (g/L)	117.6 ± 11.6	117.7 ± 11.9	0.011
Instantly before study drink			
NRS hunger score ^d^	3 [1, 5]	2 [1, 5]	0.032
NRS thirst score ^e^	5 [3, 7]	5 [3, 8]	0.012
Maternal blood glucose (mmol/l)	6.4 ± 1.3	6.4 ± 1.3	0.014

Data are mean ± SD, n (%) or median [interquartile range]. *ASD* Absolute standardized difference (an ASD of ≥0.089 is considered imbalanced between the two groups); *NRS* Numeric Rating Scale

^a^ Include asthma, arrhythmia, latent glomerulonephritis, hypertension, and positive hepatitis B surface antigen

^b^ Include hysteromyoma, ovarian cysts, dysfunctional uterine bleeding, and pelvic inflammatory disease

^c^ Include antiphospholipid syndrome, preeclampsia, gestational hypertension, acute chorioamnionitis, and thrombocytopenia in pregnancy

^d^ An 11-point scale from 0 to 10, where 0 = no hunger at all and 10 = the worst hunger

^e^ An 11-point scale from 0 to 10, where 0 = no thirst at all and 10 = the worst thirst

**Table 2:** Intrapartum variables

	Carbohydrate-rich beverage (*n =* 982)	Low-carbohydrate beverages (*n =* 971)	*p* value
Maternal variables			
Use of oxytocin during labor	460 (46.8%)	424 (49.0%)	0.18
Artificial membrane rupture	332 (33.8%)	311 (32.0%)	0.40
NRS pain score ^a^			
Before analgesia	8 [7, 10]	8 [7, 9]	0.70
10 min after analgesia	4 [3, 5]	4 [3, 5]	0.33
30 min after analgesia	2 [1, 3]	2 [1, 3]	0.94
10-cm cervical dilation ^b^	3 [3, 4] (*n =* 871)	3 [3, 4] (*n =* 865)	0.54
Dosage of labor analgesia			
Sufentanil (μg)	25 [17, 37]	26 [18, 36]	0.73
0.1% Ropivacaine (ml)	42 [30, 61]	43 [31, 59]	0.77
Lateral episiotomy ^b^	368 (42.3%) (*n =* 871)	339 (39.2%) (*n =* 865)	0.24
Anesthesia for emergent cesarean delivery	(*n =* 111)	(*n =* 106)	> 0.99
Epidural	110 (99.1%)	105 (99.1%)	
General	1 (0.9%)	1 (0.9%)	
Maximal temperature ≥ 38.0 °C	76 (7.7%)	73 (7.5%)	0.85
Estimated blood loss (ml)	240 [200, 300]	240 [200, 300]	0.72
Oral carbohydrate beverage ^c^			
Volume (ml)	584 [400, 800]	400 [100, 700]	**< 0.01**
Calorie supplied (kcal)	339 [232, 464]	84 [26, 158]	**< 0.01**
Calorie supplied (kcal/h)	64 [45, 87]	17 [5, 26]	**< 0.01**
Oral water intake (ml)	0 [0, 150]	100 [0, 310]	**< 0.01**
Intravenous fluid			
Volume (ml)	600 [500, 1000]	550 [400, 1000]	0.17
Calorie supplied (kcal)	5 [5, 9]	5 [4, 9]	0.17
Total fluid intake (ml) ^d^	1350 [979, 1830]	1250 [850, 1750]	**< 0.01**
Total calorie supplied (kcal) ^d^	348 [235, 469]	92 [31, 163]	**< 0.01**
Total calorie per hour (kcal/h)	65 [46, 88]	18 [7, 27]	**< 0.01**
Neonatal variables			
Male gender	504 (51.3%)	514 (52.9%)	0.48
Birth weight (g)	3338 ± 362	3354 ± 353	0.33
Small for gestational age ^e^	13 (1.3%)	10 (1.0%)	0.55
Large for gestational age ^f^	30 (3.1%)	36 (3.7%)	0.43

Data are mean ± SD, n (%) or median [interquartile range]. *P* values in bold indicate < 0.05. *NRS* Numeric rating scale

^a^ An 11-point scale where 0 = no pain and 10 = the worst pain

^b^ Results of parturients who gave vaginal delivery

^c^ Include oral carbohydrate-rich beverage and low-carbohydrate beverage used in current research

^d^ Sum volume of oral carbohydrate beverage, oral water, and intravenous fluid

^e^ Defined as less than the 10th cohort-specific percentile of the standardized term birthweight [31]

^f^ Defined as beyond the 90th percentile of the a standardized birthweight by gestational age and gender [32]

### Outcomes

The rate of cesarean delivery did not differ between the two groups (11.3% [111/982] with carbohydrate-rich beverage vs. 10.9% [106/971] with low-carbohydrate beverages; relative risk [RR] 1.04, 95% CI 0.81 to 1.33; *p* = 0.79). Per-protocol analysis also showed no difference (11.0% [105/955] with carbohydrate-rich beverage vs. 10.7% [101/940] with low-carbohydrate beverages; RR 1.02, 95% CI 0.79 to 1.32; *p* = 0.86; Table 3). After adjustment for total fluid intake in a logistic regression model, the differences between group remained unchanged (intention-to-treat population: adjusted RR 1.02, 95% CI 0.77 to 1.35; *p =* 0.89; per-protocol population: adjusted RR 1.01, 95% CI 0.75 to 1.35; *p =* 0.96).

**Table 3:** Outcomes

	Carbohydrate-rich beverage (*n =* 982)	Low-carbohydrate beverages (*n =* 971)	Relative risk, median difference, or mean difference (95% CI) ^a^	*p* value
**Primary outcome**				
Cesarean delivery	111 (11.3%)	106 (10.9%)	Relative risk = 1.04 (0.81, 1.33)	0.79
Cesarean delivery (PP analysis)	105 (11.0%) (*n =* 955)	101 (10.7%) (*n =* 940)	Relative risk = 1.02 (0.79, 1.32)	0.86
**Secondary outcomes**				
***Maternal outcomes***				
Duration of labor (min) ^b^	562 [445, 730] (*n =* 871)	565 [425, 725] (*n =* 865)	Median difference = 7 (−15, 30)	0.42
First labor stage ^b^	490 [360, 660] (*n =* 871)	485 [360, 660] (*n =* 865)	Median difference = 5 (− 15, 30)	0.55
Second labor stage ^b^	52 [33, 85] (*n =* 871)	50 [30, 85] (*n =* 865)	Median difference = 1 (−2, 5)	0.37
Forceps delivery	42 (4.3%)	50 (5.1%)	Relative risk = 0.83 (0.56, 1.24)	0.36
Immediately after childbirth				
NRS of hunger, score	3 [2, 5]	4 [2, 6]	Median difference = −1 (−1, 0)	**< 0.01**
NRS of thirst, score	5 [3, 7]	5 [3, 8]	Median difference = 0 (0, 0)	0.47
Maternal blood glucose (mmol/L)	7.7 ± 2.1 (*n =* 965)	7.1 ± 1.6 (*n =* 953)	Mean difference = 0.59 (0.43, 0.76)	**< 0.01**
Maternal hyperglycemia ^c^	67 (6.9%) (*n =* 965)	18 (1.9%) (*n =* 953)	Relative risk = 3.68 (2.20, 6.14)	**< 0.01**
Maternal hypoglycemia ^d^	0 (0.0%) (*n =* 965)	0 (0.0%) (*n =* 953)	–	–
***Neonatal outcomes***				
Apgar score				
< 10 at 1 min	234 (23.8%)	241 (24.8%)	Relative risk = 0.96 (0.82, 1.12)	0.61
< 10 at 5 min	16 (1.6%)	18 (1.9%)	Relative risk = 0.88 (0.45, 1.71)	0.71
Umbilical artery blood pH	7.27 [7.22, 7.32] (*n =* 960)	7.27 [7.22, 7.32] (*n =* 948)	Median difference = 0.001 (−0.004, 0.009)	0.58
pH < 7.2	164 (17.1%)	156 (16.5%)	Relative risk = 1.04 (0.85, 1.27)	0.71
pH < 7.1	12 (1.3%)	7 (0.7%)	Relative risk = 1.69 (0.67, 4.28)	0.27
Neonatal blood glucose (mmol/L)	5.5 ± 1.2 (*n =* 968)	5.1 ± 1.2 (*n =* 956)	Mean difference = 0.34 (0.24, 0.45)	**< 0.01**
Neonatal hyperglycemia ^e^	89 (9.2%) (*n =* 968)	55 (5.8%) (*n =* 956)	Relative risk = 1.60 (1.16, 2.21)	**< 0.01**
> 10.0 mmol/L	0 (0.0%) (*n =* 968)	0 (0.0%) (*n =* 956)	–	–
Neonatal hypoglycemia ^f^	10 (1.0%) (*n =* 968)	22 (2.3%) (*n =* 956)	Relative risk = 0.45 (0.21, 0.94)	**0.03**
Neonatal ward admission ^g^	22 (2.2%)	19 (2.0%)	Relative risk = 1.15 (0.62, 2.10)	0.66

Data are n (%), median [interquartile range], or mean ± SD. *P* values in bold indicate < 0.05. *PP* Per-protocol, *RR* Relative risk, *NRS* Numeric rating scale, *D* Difference

^a^ Calculated as the carbohydrate drink group vs. or minus the clear liquid drink group. Effect sizes are Hedges’ g (95% CI) or Cohen’s d (95% CI) for quantitative data and relative risk (95% CI) for categorical data

^b^ Results of parturients who gave vaginal delivery

^c^ Defined as maternal blood glucose > 11.1 mmol/L [26]

^d^ Defined as maternal blood glucose < 3.3 mmol/L.

^e^ Defined as neonatal blood glucose > 7.0 mmol/L.

^f^ Defined as neonatal blood glucose < 2.6 mmol/L.

^g^ Newborns were admitted to neonatal ward for further monitoring and/or treatment which were considered necessary by the pediatricians. Indications for neonatal ward admission included meconium-stained amniotic fluid, neonatal asphyxia, neonatal pneumothorax, fetal ventriculomegaly and mature low birth weight

After giving birth, the NRS for hunger was lower in the carbohydrate-rich group than that in the low-carbohydrate group (median 3 [interquartile range 2 to 5] points vs. 4 [2 to 6] points; median difference − 1, 95% CI − 1 to 0; *p* < 0.01). Maternal blood glucose level was higher in the carbohydrate-rich group than in the low-carbohydrate group ([7.7 ± 2.1] mmol/L vs. [7.1 ± 1.6] mmol/L; mean difference 0.59, 95% CI 0.43 to 0.76; *p* < 0.01). The incidence of maternal hyperglycemia was also higher in the carbohydrate-rich beverage group (6.9% [67/965] vs. 1.9% [18/953]; RR 3.68, 95% CI 2.20 to 6.14; *p* < 0.01). No maternal hypoglycemia occurred in both groups (Table 3).

Of all neonates, 1.6% (32/1953) developed hypoglycemia; none of them presented obvious clinical signs. For neonates with hypoglycemia, all blood glucose levels returned to normal at 30 min after glucose feeding ([4.6 ± 0.8] mmol/L) and remained normal in twice of subsequent tests in 30-min intervals ([5.6 ± 0.8] mmol/L and [5.8 ± 0.5] mmol/L, respectively). The early neonatal blood glucose level was higher in the carbohydrate-rich group than that in the low-carbohydrate group ([5.5 ± 1.2] mmol/L vs. [5.1 ± 1.2] mol/L; mean difference 0.34, 95% CI 0.24 to 0.45; *p* < 0.01). The incidence of neonatal hyperglycemia was higher (9.2% [89/968] vs. 5.8% [55/956]; RR 1.60, 95% CI 1.16 to 2.21; *p* < 0.01) whereas the incidence of neonatal hypoglycemia (1.0% [10/968] vs. 2.3% [22/956]; RR 0.45, 95% CI 0.21 to 0.94; *p* = 0.03; number needed to treat 77) was lower in the carbohydrate-rich group (Table 3).

After close monitoring for 2 h (retested every 60/30 min), glucose levels of both mothers and infants with hyperglycemia returned to normal range and remained stable. No insulin treatment was required in either mothers or neonates.

### Side effects

The incidence of vomiting during labor was lower in the carbohydrate-rich group than that in the low-carbohydrate group (9.5% [93/982] vs. 12.4% [120/971]; *p* = 0.04). No aspiration occurred in both groups (Table 4).

**Table 4:** Side effects

	Carbohydrate-rich beverage (*n =* 982)	Low-carbohydrate beverages (*n =* 971)	*p* value
Vomiting during labor	93 (9.5%)	120 (12.4%)	**0.04**
Vomiting during cesarean delivery ^a^	16 (14.4%) (*n =* 111)	16 (17.8%) (*n =* 106)	0.52
Aspiration during cesarean delivery	0 (0.0%)	0 (0.0%)	–
Bradycardia during labor ^b^	0 (0.0%)	0 (0.0%)	–
Hypotension during labor ^c^	0 (0.0%)	0 (0.0%)	–

Data are n (%). Numbers in square brackets indicate patients with missing data. *P* values in bold indicate < 0.05

^a^ Results of parturients who received cesarean section

^b^ Defined as heart rate < 60 beats per minute

^c^ Defined as systolic blood pressure < 90 mmHg or a decrease of > 30% from baseline

## Discussion

Our results show that carbohydrate-rich beverage compared with low-carbohydrate beverages for women during epidural labor analgesia did not reduce cesarean delivery, nor did it alter duration of labor and rate of instrumental delivery. However, carbohydrate-rich beverage relieved maternal hunger more effectively and decreased neonatal hypoglycemic episodes after birth. Higher blood glucose levels and more hyperglycemic episodes of both mothers and neonates were found in the carbohydrate-rich beverage group.

The policy of restricting food intake during labor is a common practice across many birth settings, with parturients being fasted or only allowed to have sips of water or ice chips, especially in the United States [34]. However, the rule is being challenged, because fluid loss and calorie consumption are high during parturition, as are the body’s requirements for hydration and nutrition [35]. Indeed, the basal metabolic rate is increased during pregnancy, and labor further increases the energy demand for reasons including regular uterine smooth muscle cell contractions, abdominal muscle contractions, and forced Valsalva maneuvers when pushing [9]. Ketosis, which occurs when glycogen stores are depleted and the body metabolizes fat for energy, is common during labor [36]. Ketoacids can readily cross the placenta and reach the fetus. And, an association between prolonged labor and ketone production has been demonstrated [36]. Therefore, nutritional support during childbirth is necessary.

Maltodextrin is a compound of maltose and dextrin (chain of glucose polymers) made from partial hydrolysis of corn starch and has a dextrose equivalent less than 20 [37]; it is frequently used as a carbohydrate supplement in sports nutrition in the hope of maximizing glycogen storage [38]. For athletes during prolonged moderate-to-high intensity exercise, ingestion of carbohydrate solution can significantly reduce lipolysis, ketosis and protein degradation, and improve endurance performance [11, 39]. There is a paucity of data regarding energy requirements of laboring women, and studies showed that a calorie intake of 47 kcal/h prevents parturients from ketosis [19]. In our study, the calorie supply per hour during labor was 65 kcal/h in the carbohydrate-rich group and 18 kcal/h in the low-carbohydrate group, respectively. Although the calorie intake in the carbohydrate-rich group would be high enough to prevent ketosis as well, we could find rate differences of neither cesarean delivery nor forceps delivery. Similar results were also reported by others comparing low-carbohydrate drinks vs. none-carbohydrate drink or food intake vs. fasting/water drink [14, 17–20, 40, 41]. Thus, taken together, it seems that the content of carbohydrate supplementation does not change mode of delivery.

In addition to supplying hydration and nutrition, carbohydrate-containing beverage may also produce psychological effects in laboring women. Self-regulated oral intake decreases the stress level of women and provides the sense of control and self-confidence [42]. There is a growing interest in the use of energy-rich carbohydrate beverage as part of the strategy of enhanced recovery after surgery, which aims at ameliorating weakness and discomfort (such as hunger, thirst, chill, etc.) and improving clinical outcomes when food-restriction is necessary [43]. We also found that, compared with low-carbohydrate beverages, the carbohydrate-rich beverage significantly decreased the subjective hunger score after childbirth although clinical importance of this difference still needs to be clarified.

For the fetus, there is a constant glucose infusion via facilitated diffusion across the placenta according to the maternal-to-fetal glucose concentration gradient [44]. At birth, fetal blood glucose is about 70–80% of maternal glucose level [45], which was consistent with our data. There is no consensus regarding optimal timepoint for neonatal glucose monitoring. In previous studies, blood glucose was tested within the first 180 min of newborns, the expected timing of glucose nadir [27, 46]. In the present study, neonatal blood glucose was tested from immediate birth to 90 min later with three 30-min intervals. We also found that blood glucose level and hyperglycemia rate in both mothers and neonates were higher in women with carbohydrate-rich beverage than in those with commercial low-carbohydrate beverages. Hyperglycemia is a common metabolic abnormality in low-birthweight, preterm, and critically ill newborns [47]. Hyperglycemia of > 10 mmol/L in extremely preterm infants is associated with high mortality [30]. Persistent hyperglycemia in newborns may induce dehydration, ketosis, weight loss, and susceptibility to infection [47]. Lemelman et al. [48] recommend insulin treatment when blood glucose is higher than 13.8 mmol/L. In the present study, no newborn had blood glucose > 10 mmol/L; all elevated blood glucose returned to normal in subsequent tests without intervention. On the other hand, our results showed that rate of neonatal hypoglycemia was reduced in mothers with carbohydrate-rich beverage. In clinical practice, neonatal blood glucose monitoring is recommended for those at risk (such as preterm newborns, small for gestational age, and infants of diabetic mothers) or with symptomatic hypoglycemia, but is not routinely performed in healthy infants [49]. Given the fact that 1.6% of newborns without risk factors developed asymptomatic hypoglycemia in our study, and concerns of potential harmful effects of transient new-born hypoglycemia [27], carbohydrate-rich beverage could be an appropriate source of energy to solve the issue during childbirth.

In the present study, the overall rate of vomiting during labor was 10.9%, lower than those reported in previous studies (range from 15.6 to 37%) [17, 40, 50, 51]. Furthermore, the rate of vomiting was lower in the carbohydrate-rich group than in the low-carbohydrate group in our finding. It might be related to the study population, or sample size, or consumed calories that, in the past, the incidence difference of vomiting in parturients with energy-drinks vs. placebo-drink or other fluids/foods was not statistically significant [14, 18, 41]. This might be attributed to the differences in study populations and clinical practice. Given that vomiting can lead to discomfort, electrolyte imbalance, dehydration and even aspiration, a lower risk of vomiting may be another favorable effect of oral energy-rich solution although further studies are required to confirm this. However, no aspiration occurred in our patients, possibly because that labor epidural analgesia does not worsen gastric emptying [52], most emergent cesarean cases were performed under epidural anesthesia, and measures for full-stomach were adopted during general anesthesia. Further studies are needed to confirm the safety of oral liquid intake during labor.

The strength of our pragmatic trial includes a multicenter design and a sufficient sample size. There are also several limitations. First, as an open-label trial, there existed possibility for performance or assessment bias. To minimize the potential bias, maternal sensation of hunger/thirst and maternal/neonatal blood glucose were assessed by investigators who were not involved in clinical decision-making. Second, parturients in the carbohydrate-rich group were encouraged to take the beverage and turned out to have more hydration, which may produce bias [53], although adjustment with hydration volume did not change our results. Third, we did not record the dietary intake prior to labor and the time interval since last meal which might influence the outcomes. But strict randomization and large sample size should have balanced these factors between groups. Fourth, the point-of-care glucometer is not the gold standard measurement to determine blood glucose levels and the accuracy may be challenged although a study had confirmed that Accu-Chek® glucometer can be used effectively in neonatal settings to detect hypoglycemia [54].

## Conclusions

For primigravidae undergoing epidural analgesia during labor, ingestion of a carbohydrate-rich beverage compared with low-carbohydrate beverages does not reduce cesarean delivery nor forceps delivery, but relieved maternal hunger and reduced incidence of neonatal hypoglycemia after birth. On the other hand, carbohydrate-rich beverage also increased blood glucose level and hyperglycemic episodes of both mothers and neonates. Optimal dose of carbohydrate supplementation during labor analgesia remains to be determined.

## Supplementary Information

**Additional file 1: File S1.** Study protocol.

**Additional file 2: Table S1.** Enrollment by site.

## Data Availability

The datasets generated and/or analyzed during the current study are not publicly available due to individual privacy issues but are available from the corresponding author on reasonable request.

## Abbreviations

RR: Risk ratio
CI: Confidence interval
NPRS: Numeric Pain Rating Scale
NRS: Numeric Rating Scale
ASD: Absolute standardized difference
