# Supplementary material for: Effect of a carbohydrate-rich beverage on rate of cesarean delivery in primigravidae with epidural labor analgesia: a multicenter randomized trial
Source: BMC Pregnancy Childbirth. 2022 Apr 19;22:339. doi: 10.1186/s12884-022-04659-2 (PMC9019984; doi:10.1186/s12884-022-04659-2)
Supplement: Supplementary file 1 — Additional file 1: File S1. Study protocol. [file 12884_2022_4659_MOESM1_ESM.docx]

**Additional file 1: Study protocol**

**Effect of a carbohydrate-rich beverage on rate of cesarean delivery in parturients with epidural labor analgesia: a multicenter randomized trial**

**Study Design:** Ting Ding, MD, Yuan Qu, MD, Dong-Xin Wang, MD, PhD

**Name of institute:** Department of Anesthesiology and Critical Care Medicine, Peking University First Hospital, Beijing 100034, China

**Version of protocol:** V 1.5

**Time of last revision:** July 2017

**Summary of revision of the protocol**

|  | Version | Date of version | Drafter/  reviser | Contents of revision |
| --- | --- | --- | --- | --- |
| 1 | 1.4 | Jun. 9, 2017 | T.D., Y.Q., D-X.W. | (This was the first version approved by the ethics committee before recruiting patients). |
| 2 | 1.5 | Jul. 2,  2017 | T.D., Y.Q., D-X.W. | 1) Expanded the estimated sample size to 2000 in nine study sites (clause 4.2).  2) Clarified that “this trial will be conducted in 9 obstetric-led maternity units in China” (clause 4.3). |

**Contents**

1. Background 4

2. Purposes of the study 4

3. Participants 4

4. Study design 6

5. Randomization and blinding 6

6. Interventional protocol 7

7. Data collection 8

8. Outcome assessment 9

9. Adverse events and management 9

10. Severe adverse events 10

11. Unmask of blindness 11

12. Database management 11

13. Statistical Analysis 11

14. Quality control and quality assurance 13

15. Ethics requirement and written informed consent 14

16. Study termination 14

17. Documents preservation 15

18. Declaration of interests 15

19. Reference 15

**1. Background**

The recommendations to particularly restrict oral intake in laboring women came from Mendelson’s work in the 1940s, which reported an increased risk of aspiration of gastric contents in the event that general anesthesia was required [1]. However, from a physiological perspective, it is crucial to supply sufficient energy and hydration for women during labor, which is similar to performing moderate, continuous aerobic exercise [2]. Starvation decreases hepatic glycogen stores, induces a state of insulin resistance [3, 4], and impairs cardiovascular function [5]. Adequate maternal calorie supply during labor is fundamental to ensure effective uterine contractions [6]. Energy depletion during labor can stimulate alterations in the acid–base balance of the myometrium, provoke a reduction in contractility and prolong labor [7], as well as increase the probability of a cesarean delivery [6]. With the development of obstetric anesthesia, pulmonary aspiration during general anesthesia for cesarean delivery has declined dramatically in recent years [8]. The American Society of Anesthesiologists (ASA) and the American College of Obstetricians and Gynecologists (ACOG) prudently recommended that “the oral intake of modest amounts of clear liquids (e.g., water, black coffee, and sports drinks) may be allowed for uncomplicated laboring patients”; however, “solid foods should be avoided in laboring patients” [9, 10]. For more than 15 years, the debate regarding traditional oral intake restriction regimens during labor has never been ceased.

Labor has been described as a prolonged exercise [11]. As there is still a general lack of knowledge concerning the energy expenditure during labor, studies focusing on this field often reference recommendations in sports medicine. According to the advice of the American College of Sports Medicine, athletes who are undertaking intense, moderate and intermittent exercise should ingest carbohydrate solutions; this can not only enhance the capacity of performance but also decelerate the occurrence of exhaustion [12]. Oral rehydration solutions with carbohydrate polymers have been designed to reduce the negative effects of fasting [13]. Maltodextrin carbohydrate polymer allows for the absorption of sufficient glucose to stimulate insulin secretion to restore glycogen stores, similar to the consumption of a meal [14]. Epidural analgesia is frequently used during labor to relieve pain intensity and attenuates stress response [15]. According to the ASA and ACOG guidelines, the Chinese Society of Anesthesiologists Task Force on Obstetric Anesthesia encourages parturients to ingest an energy-dense, low residue beverage during epidural labor analgesia [16]. At present, clear liquids (mostly commercial sports drinks) are allowed in parturients with epidural labor analgesia in some Chinese hospitals [17]. However, these liquid beverages only provide limited amount carbohydrate. Furthermore, available studies have compared the effectiveness of carbohydrate drink to either fasted parturients or a carbohydrate-free placebo of water or flavored water [18]. None of these studies included common, commercial sports drinks with lower carbohydrate content and compared their effects against beverages with a higher carbohydrate content. We speculated that, for women in labor with epidural analgesia, the intake of carbohydrate-rich drink might improve mothers’ performance and reduce cesarean delivery.

**2. Purposes of the study**

To observe the impact of carbohydrate-rich drink on the incidence of cesarean delivery in laboring women with epidural analgesia.

**3. Participants**

***3.1 Inclusion Criteria***

3.1.1 Primipara with a single cephalic pregnancy beyond 37 weeks.

3.1.2 Admitted to the delivery ward for vaginal birth and request epidural labor analgesia.

***3.2 Exclusion Criteria***

Patients who meet any of the following criteria will be excluded.

3.2.1 Age <18 or >34 years.

3.2.2 Comorbid diabetes mellitus or gestational diabetes mellitus.

3.2.3 Suspected fetal abnormalities by prenatal ultrasonography.

3.2.4 Presence of contraindications to epidural analgesia (including history of infectious disease of the central nervous system, history of spinal or intraspinal disease, systemic infection, skin or soft tissue infection at the site of puncture, and coagulopathy).

3.2.5 Other conditions that are considered unsuitable for study participation.

***3.3 Criteria of drop-out***

3.3.1 Exit by participants themselves (i.e., withdraw consents).

3.3.2 Loss to follow-up.

3.3.3 Ordered to exit by the investigators (poor compliance, occurrence of severe complications, or occurrence of severe adverse events).

For drop-out cases, detailed reasons should be recorded. The primary therapeutic effects recorded in the last time will be regarded as the final results. The cases will be included in the intention-to-treat analysis. The Case Report Forms of these cases will be kept for future reference.

***3.4 Criteria of elimination***

Included patients will be excluded if they meet any of the following criteria:

3.4.1 Withdraw consents before intervention.

3.4.2 Do not receive any designated oral beverage.

3.4.3 No assessment result of the primary outcome.

The causes of elimination should be explained. The case will be excluded from the intention-to-treat analysis. The case report forms will be preserved for reference.

***3.5 Criteria of study interruption***

Study will be discontinued if any of the followings happens.

3.5.1 Severe safety problems.

3.5.2 Severe mistake in the protocol.

3.5.3 Support withdrawn by the sponsor or management reasons.

3.5.4 Study cancelled by the administrative department.

Study suspension may be temporary or permanent. In case the study is suspended, all records will be filed for future reference.

**4. Study design**

***4.1 Type of the study***

This is a multicenter, randomized controlled trial with two parallel arms.

***4.2 Sample size calculation***

In a pilot trial of our own, the rate of cesarean delivery was 8% of parturients with carbohydrate drink and 12% of those with clear liquid drink. We assumed similar rates in the present study. With the significance level set at 0.05 (two-sided) and power set at 80%, the sample size required to detect difference was 1760 parturients. Considering a drop-out rate of about 10%, we plan to enroll 2000 parturients. Sample size calculation was performed with the Stata 10.0 software (Stata Corp. LP, College Station, TX, USA).

***4.3 Study center***

This trial will be conducted in 9 obstetric-led maternity units in tertiary hospitals in China. Nine hospitals expected to participate in this study are Peking University First hospital; Woman's Hospital of Nanjing Medical University; Tangshan Maternity and Child Health Care Hospital; Foshan Maternal and Child Health Hospital; Gansu Provincial Maternity and Child Care Hospital; Urumqi Women and Child Health Care Hospital; Linyi people's hospital; Anhui Women and Child Health Care Hospital; Women's Hospital of Zhejiang University.

**5. Randomization and blinding**

***5.1 Randomization principle***

5.1.1 A biostatistician, who is independent of data management and statistical analyses, generates random numbers in a 1:1 ratio with a block size of 4 using the SAS 9.2 software (SAS Institute, Cary, NC). Randomization is stratified according to the study centers.

5.1.2 Study beverages (carbohydrate-rich beverage and commercially available low-carbohydrate beverages) are provided according to the randomization results by an anesthesiologist who does not participate in the other part of the study.

5.1.3 The results of randomization are sealed in opaque, sequentially numbered envelopes and stored at the sites of investigation.

***5.2 Blinding***

Because of the apparent differences in oral drink appearance, study participants, healthcare providers, and investigators are not masked from the study group allocation; however, cesarean delivery rate, maternal sensation of hunger and thirst, and maternal and neonatal blood glucose will be recorded or assessed by outcome assessors who are not involved in perinatal care.

***5.3 Emergency unblinding***

5.3.1 In emergency cases (such as sudden deterioration of parturients’ condition), clinicians and investigators can ask for stopping study intervention.

5.3.2 The reasons leading to interruption of study intervention will be recorded in the case report forms.

**6. Interventional protocol**

***6.1 Epidural labor analgesia management***

6.1.1 As a routine practice, cervical dilatation is assessed at 4-hour intervals after delivery room admission. Epidural labor analgesia is initiated when the cervix was dilated to 1 cm or more.

6.1.2 Before the implement of analgesia, an intravenous line is established with Ringer’s lactate solution (50 ml/h; Na^+^ 130 mmol/L, K^+^ 4 mmol/L, 0.009 kcal/ml).

6.1.3 After confirming the position of the epidural catheter inserted between L2-3 or L3-4 intervertebral space, a 10 ml mixture of 0.1% ropivacaine (AstraZeneca AB, Södertälje, Sweden) plus 0.5 μg/ml sufentanil (EuroCept BV, Ankeveen, Netherlands) is administered as a loading dose; an additional 5 ml mixture is administered 10 minutes later if the numeric rating scale (NRS, an 11-point scale where 0=no pain and 10=the worst pain) of pain remained ≥4.

6.1.4 An intermittent epidural bolus pump (ZZB-II; Jiangsu Aipeng Medical Science and Technology Company Ltd., Nantong, China) is attached 30 minutes later, which is established with 200 ml mixture of 0.07% ropivacaine plus 0.45 μg/ml sufentanil and programmed to deliver 6-ml boluses with a lockout interval of 20 minutes and a background infusion of 4 ml/h. The maximal dose limit is 26 ml/h.

6.1.5 The PCEA pump is stopped at the end of the third stage of labor.

***6.2 Study intervention***

6.2.1 Study intervention will be administered during epidural labor analgesia.

6.2.2 Parturients in the carbohydrate-rich group are provided with an oral carbohydrate-rich solution [Outfast®: 285 mOsm/kg, 14.1% carbohydrate (maltodextrin 100g/L, fructose 23.9g/L, glucose 16.9g/L), Na^+^ 2.0 mmol/L, K^+^ 4.9 mmol/L, 0.58 kcal/ml; Yichang Humanwell Pharmaceutical Co, Ltd, Hubei, China] and are advised to drink as will during labor.

6.2.3 Parturients in the low-carbohydrate group are advised to drink commercially available low-carbohydrate beverages as will (Pocarisweat®: 0.26 kcal/ml, Otsuka Pharmaceutical Co. Ltd, Tokyo, Japan; Gatorade®, 0.24 kcal/ml, PepsiCo, Chicago, IL, USA; or Mizone®: 0.21 kcal/ml, Danone, Guangzhou, Guangdong, China).

6.2.4 All participants have free access to water, but solid foods are not permitted.

6.2.5 Dietary intake prior to labor analgesia and after the third stage of labor are not interfered.

***6.3 Intrapartum care***

6.3.1 In the delivery ward, maternal vital signs are monitoring every 1 to 2 hours but more frequently when necessary. Continuous external fetal heart rate monitoring and/or tocodynamometry are performed as indicated.

6.3.2 Obstetric managements such as oxytocin administration, forceps assistance, and cesarean delivery are decided by the attending obstetricians and midwives. Cesarean delivery is decided according to Chinese guidelines [19].

6.3.3 In case of emergency cesarean delivery, epidural anesthesia is performed through the indwelling epidural catheter or, otherwise, combined spinal-epidural anesthesia is performed. For parturients requiring general anesthesia, a rapid sequence induction with endotracheal intubation is performed. Cricoid pressure is applied continuously from loss of protective airway reflexes until accomplishment of endotracheal intubation and cuff inflation. Antacid prophylaxis and nasogastric tube are not routinely used.

6.3.4 All newborns are observed for 2 hours in the delivery room before being transferred to the postpartum ward.

***6.4 Postpartum care***

The rooming-in policy is the standard of care in all participating centers. Breastfeeding is encouraged whenever possible.

**7. Data collection**

***7.1 Prepartum data***

7.1.1 Demographic data, including gender, date of birth, and body mass index.

7.1.2 Diagnosis and medical history, including duration of gestation, gravidity, prepartum comorbidities, comorbid obstetric disease, medical treatment, history of allergy to food and drugs, and prepartum hemoglobin

7.1.3 Subjective discomfort (degree of hunger and thirst) is assessed using the Numeric Rating Scale (NRS, an 11-point scale where 0 indicated no hunger/thirst and 10 indicated the most severe hunger/thirst) at the initiation of epidural labor analgesia.

7.1.4 Maternal blood glucose level is tested using a blood glucose monitor (Accu-Chek®, Roche, Germany) at the initiation of epidural labor analgesia.

***7.2 Intrapartum data***

7.2.1 Maternal variables, including oxytocin requirements, artificial membrane rupture, dosage of labor analgesia, duration of labor, episiotomy, mode of delivery, maximal temperature, estimated blood loss, volume of oral intake and volume of intravenous fluid.

7.2.2 Pain severity is assessed using the Numeric Rating Scale (NRS, an 11-point scale where 0 indicated no pain and 10 indicated the most severe pain) before analgesia, at 10 and 30 minutes after analgesia, and at full cervical dilation.

7.3.3 Calculate data, including total fluid intake (oral and intravenous) and calories supplied by oral and intravenous fluid.

***7.3 Postpartum data***

7.3.1 Neonatal variables, including gender, birth weight, Apgar scores at 1 and 5 minutes after birth, and neonatal ward admission. Neonatal blood glucose level is tested instantly after birth using a blood glucose monitor (Accu-Chek®, Roche, Germany), and will be retested every 30 minutes when necessary.

7.3.2 Maternal blood glucose level is tested instantly after giving birth using a blood glucose monitor (Accu-Chek®, Roche, Germany) and will be retested every 30 minutes when necessary.

**8. Outcome assessment**

***8.1 Primary outcomes***

The rate of cesarean delivery.

***8.2 Secondary outcomes***

8.2.1 Duration of labor.

8.2.2 Rate of forceps delivery.

8.2.3 The NRS score of hunger/thirst.

8.2.4 Maternal blood glucose after giving birth.

8.2.5 1- and 5-minute Apgar scores.

8.2.6 The umbilical artery pH value after birth.

8.2.7 Neonatal blood glucose after birth.

8.2.8 The rate of neonatal ward admission.

**9. Adverse events and management**

***9.1 Definition***

An adverse event indicates any unpredictable, unfavorable medical event that is associated with any medical intervention and occurred during the study period. It can be related to the study beverage administration or not. It can manifest as any uncomfortable signs (including abnormal laboratory findings), symptoms or transient morbidity. In the present study, adverse events are monitored from the initiating of epidural labor analgesia until 2 hours postpartum in the delivery room (before being transferred to the postpartum ward).

***9.2 Monitoring and record of adverse events***

9.2.1 In delivery ward, intermittent maternal vital sign monitoring (every 1-2 hours) will be performed.

9.2.2 Continuous maternal vital sign monitoring will be performed from the initiating of epidural labor analgesia to 30 minutes after the administration of loading dose.

9.2.3 Continuous external fetal heart rate monitoring and/or tocodynamometry will be performed as indicated.

9.2.4 Continuous maternal vital sign and external fetal heart rate monitoring will be performed during the second stage of labor.

***9.3 Diagnosis and management of predictable adverse events***

9.3.1 Bradycardia is defined as HR <60 beats per minute, and is managed with atropine 0.2-0.4 mg (i.v., repeat in 5-10 minutes if necessary).

9.3.2 Hypotension is defined as systolic blood pressure <90 mmHg or a decrease of >30% from baseline and is managed with fluid infusion and/or vasopressors.

9.3.3 Maternal hypoglycemia is defined as blood glucose <3.3 mmol/L [20] and is managed with oral or intravenous glucose.

9.3.4 Maternal hyperglycemia is defined as blood glucose >11.1 mmol/L [21]; insulin will be administered prudently when considered necessary.

9.3.5 Neonatal hypoglycemia is defined as blood glucose <2.6 mmol/L [22, 23], and will be treated with 5% glucose oral feeding (10-ml) [24].

9.3.6 Neonatal hyperglycemia is defined as blood glucose >7.0 mmol/L. In term newborns, hyperglycemia is generally regarded as part of stress response and not managed unless blood glucose >10 mmol/L [25]. Considering the harmful effects of insulin-induced hypoglycemia, infants with hyperglycemia will be managed with a wait-and-see strategy in all participating centers.

9.3.7 Maternal nausea and vomiting during cesarean delivery is managed with antiemetics, including dexamethasone, 5-HT3 receptor antagonists (tropisetron).

9.3.8 Pulmonary aspiration is defined as the inhalation of oropharyngeal or gastric contents into the respiratory tract. Initial treatment of a significant aspiration consists of oropharyngeal or endotracheal suctioning, administration of bronchodilators for bronchospasm, and supplemental O2. Bronchoscopy may be used as indicated. Mechanical ventilatory support with positive end-expiratory pressure may be necessary if hypoxemia is severe. Empirical antibiotics will be administrated when considered necessary. Other management including pulse oximetry monitoring and chest radiography will be performed according to routine practice [26].

9.3.9 For parturients with adverse events that are suspected to be related to the study beverage, healthcare team members should provide management as above and according to routine practice; they can discontinue study beverage administration if considered necessary and inform the investigators.

***9.4 Record***

9.4.1 Any adverse event should be documented, including occurrence, type/diagnosis, time of diagnosis, management, duration of persistence, and sequelae.

9.4.2 All types of adverse event which occur to parturients in the trial will be reported as soon as possible after research staff become aware of the event.

9.4.3 Any adverse event should be followed up until it is completely resolved, or therapy terminated.

**10. Severe adverse events**

***10.1 Definition***

A severe adverse event indicates any unpredictable medical events that lead to death, threat to life, prolonged length of stay in hospital, persistent disability or vital organ dysfunction, or other severe results. In the present study, severe adverse events are monitored from the initiating of epidural labor analgesia until 2 hours postpartum in the delivery room (before being transferred to the postpartum ward).

***10.2 Management and report***

10.2.1 In case of any severe adverse events, stop study beverage intake and start treatment immediately.

10.2.2 In case of any severe adverse events, apart from active treatment and rescue, inform the principal investigator and the Ethics Committee within 24 hours by telephone and in written report.

10.2.3 In case of study related death, immediately stop the clinical trial, report the event to the Ethics Committee as soon as possible, record in detail and carefully preserve the related documents.

10.2.4 Any severe adverse event must be followed up until it is completely resolved, or the treatment is terminated.

**11. Unmask of blindness**

11.1 After each trial patient’s study is completed and all data have been inputted into the database and checked without mistakes, the database will be blindly reviewed, and the analysis sets will be defined.

11.2 Unblinding will be performed after the database is locked. The database will be sent to a biostatistician for statistical analysis.

**12. Database management**

12.1 Original data will be recorded in the case report forms promptly and accurately. All recorded data will be kept confidentially.

12.2 The completed case report forms will be checked by a study coordinator who is authorized by the principal investigator. Amendment will be made when necessary.

12.3 After accomplishment of data entry and check using a study-specific REDCap database [27], all case report forms will be stored in sequence.

12.4 Data management and data monitoring will be inspected by the Clinical Research Ethics Committee of Peking University First Hospital.

**13. Statistical analysis**

***13.1 Analysis populations***

13.1.1 Intention-to-treat population

This population includes all patients who are randomized into the study and completed expected follow-up. Exclusion of participants will be performed in a minimal and reasonable way according to the intention-to-treat principle. All efficacy evaluations will be performed in the intention-to-treat population.

13.1.2 Per-protocol population

This population includes parturients who are randomized into the study and consistent with all the following conditions: (1) ingested study beverage, (2) do not intake any prohibited foods, and (3) complete expected follow-ups. The primary outcome evaluation will also be performed in the per-protocol population.

13.1.3 Safety analysis population

This population includes all parturients who are randomized into the study and provided follow-up data for safety analysis. All safety data collected from participants including adverse events and laboratory test results will be evaluated and analyzed.

***13.2 General principles***

13.2.1 Numeric variables are described as mean ± standard deviation, median (interquartile range). Categorical variables are described as number of cases (percentage).

13.2.2 For each hypothesis, two-tailed tests will be used in all statistical analysis, P<0.05 will be considered statistically significant (unless otherwise indicated). For the treatment-by-covariate interaction in predefined subgroup analyses, a P<0.10 will be defined as statistically significant.

13.2.3 Statistical analysis will be performed with the SPSS 25.0 software package (IBM SPSS, Chicago, IL) and SAS 9.2 software (SAS Institute, Cary, NC).

***13.3 Patient recruitment and drop-out status***

The status of patient recruitment and drop-out will be summarized and listed. Comparison of the overall drop-out rate between the two groups will be performed with Chi-Square test.

***13.4 Demographics and baseline characteristics***

13.4.1 Demographic information and baseline characteristics will be presented.

13.4.2 The randomized groups are descriptively compared on all baseline variables using the absolute standardized differences (ASDs), defined as the absolute difference in means, mean ranks, or proportions divided by the pooled standard deviation. Baseline variables with an absolute standardized difference ≥1.96×$\sqrt{\text{(n1+n2)/(n1×n2)}}$ will be considered imbalanced and adjusted for in all analyses, using the formula published by Austin [28].

***13.5 Intrapartum variables***

Numeric variables will be analyzed using the independent-samples t test or Mann-Whitney U test; categorical variables will be analyzed using the chi square test, continuity correction chi square test or Fisher exact test. Missing data will not be replaced.

***13.6 Efficacy evaluation***

13.6.1 Primary endpoint:

13.6.1.1 The incidence of cesarean delivery will be calculated. Comparation between groups will be performed using Chi-Square test, with difference between groups expressed as relative risk (95% CI). The upper limit of the 95% CI of less than 1 is regarded as statistical superiority.

13.6.1.2 The interactions between treatment effect and predefined factors will be assessed separately with logistic regression models. The predefined factors include study site, age, body mass index, prepartum anemia, medical comorbidity, obstetric disease, and gynecological disease. The relative risk (and 95% CI) for each subgroup and the P values of treat-by-covariate interactions will be displayed in a forest plot.

13.6.2 Secondary endpoints:

13.6.2.1 Categorial variables (rate of forceps delivery, the rate of neonatal ward admission) between groups will be compared using Chi-Square test, continuity correction Chi-Square test, or Fisher’s exact test. Missing data will not be replaced.

13.6.2.2 Numerical variables (maternal and neonatal blood glucose after giving birth, 1- and 5-minute Apgar scores and the umbilical artery pH value) between groups will be compared using independent sample t-test or Mann-Whitney u test. Missing data will not be replaced.

13.6.2.3 Ranked data (NRS score of hunger/thirst) will be compared using Mann-Whitney u test. Missing data will not be replaced.

13.6.2.4 Relative risk (95% CI) for categorial variables, mean/median difference (95% CI) for numerical variables/ranked data will be calculated.

13.6.3 Safety outcomes

13.6.3.1 Describe the occurrence of (severe) adverse events in each group.

13.6.3.2 Describe the management of (severe) adverse events when appropriate.

13.6.3.3 The rates of (severe) adverse events and/or managements between the two groups will be compared with Chi-Square test, continuity correction Chi-Square test, or Fisher exact test.

13.6.3.4 Missing data will not be replaced.

***13.7 Conduct of statistical analysis***

Statistical analysis will be performed in the Department of Biostatistics of Peking University First Hospital.

**14. Quality control and quality assurance**

14.1 Before the study, investigators are trained to follow the study protocol.

14.2 Study protocol will be thoroughly explained to all health-care team members before the start of the study.

14.3 The monitors and instruments that are used during the study period will be checked and adjusted regularly to guarantee data accuracy.

14.4 All expected and unexpected findings will be documented promptly and correctly in order to guarantee the reliability of the values.

14.5 The study coordinators of each participating center who are authorized by the principal investigator will monitor the conduct of the study and guarantee that the study protocol is strictly adhered throughout the study period.

14.6 Data analysis will be performed by the biostatisticians and investigators.

14.7 Any conclusions must be derived from the original data.

**15. Ethics requirement and written informed consent**

***15.1 Ethics Committee***

The study protocol must be approved by the Ethics Committee before the study can be started. The investigators must strictly follow the Helsinki Declaration and China's relevant clinical trial management regulations. The principal investigator is responsible to report the status and the progress of the study to the Ethics Committee.

***15.2 Written informed consent***

Investigators responsible for recruiting participants must have been trained and qualified by the principal investigator. For each potential participant, investigators are responsible to fully explain the purpose, procedures and possible risks of this study in a written form manner. The investigators must let every potential participant know that she has the right to withdraw consent from the study at any time. Every potential participant must be given a written informed consent. Every participant or the authorized surrogate of the participant must sign the consent before she can be enrolled in the study. The written informed consents will be kept as a part of the clinical trial documents.

***15.3 Privacy and confidentiality***

15.3.1 During the study period, the collected data from participants are labeled with special recruitment numbers and acronyms of names.

15.3.2 All personal information of the participants will be kept confidential. The filing cabinets storing the study documents will be locked. Apart from the study investigators, only authorized inspectors from the Research Office of Peking University First Hospital or members from the Ethics Committee of Peking University First Hospital are allowed to access the information after obtaining consents from the participants.

15.3.3 Results of the study will be published as scientific articles. But all personal data (including name and age, etc.) are strictly confidential.

**16. Study termination**

16.1 In case that severe adverse events or serious quality problem occur during the study period, the study will be stopped. A report will be sent to the Ethics Committee. Restart of the study will need an approval from the Ethics Committee.

16.2 The study will be terminated after accomplishment of required patient recruitment and data collection. Decision will be made by the principal investigator.

**17. Documents preservation**

Investigators should carefully preserve all documents and data of clinical trial according to the Good Clinical Practice requirement.

**18. Declaration of interests**

This study is funded by Chinese Society of Cardiothoracic and Vascular Anesthesiology [20170928014]. TD and D-XW report receiving lecture fees and travel expenses for lectures given at domestic academic meetings from Yichang Humanwell Pharmaceutical Co Ltd, China. The other authors reported no conflict of interests.

**19. References**

1. Robinson M, Davidson A. Aspiration under anaesthesia: Risk assessment and decision-making. Contin Educ Anaesthesia, Crit Care Pain. 2014;14:171–5.

2. Montain SJ. Hydration recommendations for sport 2008. Curr Sports Med Rep. 7:187–92.

3. Rothman DL, Magnusson I, Katz LD, Shulman RG, Shulman GI. Quantitation of hepatic glycogenolysis and gluconeogenesis in fasting humans with 13C NMR. Science. 1991;254:573–6.

4. Awad S, Constantin-Teodosiu D, Macdonald IA, Lobo DN. Short-term starvation and mitochondrial dysfunction - a possible mechanism leading to postoperative insulin resistance. Clin Nutr. 2009;28:497–509.

5. Breuer J-P, von Dossow V, von Heymann C, Griesbach M, von Schickfus M, Mackh E, et al. Preoperative oral carbohydrate administration to ASA III-IV patients undergoing elective cardiac surgery. Anesth Analg. 2006;103:1099–108.

6. Eslamian L, Marsoosi V, Pakneeyat Y. Increased intravenous fluid intake and the course of labor in nulliparous women. Int J Gynaecol Obstet. 2006;93:102–5.

7. Pierce SJ, Kupittayanant S, Shmygol T, Wray S. The effects of pH change on Ca(++) signaling and force in pregnant human myometrium. Am J Obstet Gynecol. 2003;188:1031–8.

8. Lewis G, ed. Saving mothers’ lives: reviewing maternal deaths to make motherhood safer—2003-2005. The seventh report on confidential enquiries into maternal deaths in the United Kingdom. London: CEMACH, 2007.

9. Practice Guidelines for Obstetric Anesthesia: An Updated Report by the American Society of Anesthesiologists Task Force on Obstetric Anesthesia and the Society for Obstetric Anesthesia and Perinatology. Anesthesiology. 2016;124:270–300.

10. ACOG Committee Opinion No. 441: Oral Intake During Labor. Obstet Gynecol. 2009;114:714.

11. Dawood F, Dowswell T, Quenby S. Intravenous fluids for reducing the duration of labour in low risk nulliparous women. Cochrane database Syst Rev. 2013;:CD007715.

12. Cermak NM, van Loon LJC. The use of carbohydrates during exercise as an ergogenic aid. Sports Med. 2013;43:1139–55.

13. Bilku DK, Dennison AR, Hall TC, Metcalfe MS, Garcea G. Role of preoperative carbohydrate loading: a systematic review. Ann R Coll Surg Engl. 2014;96:15–22.

14. Kratzing C. Pre-operative nutrition and carbohydrate loading. Proc Nutr Soc. 2011;70:311–5.

15. Neumark J, Hammerle AF, Biegelmayer C. Effects of epidural analgesia on plasma catecholamines and cortisol in parturition. Acta Anaesthesiol Scand. 1985;29:555–9.

16. Chinese Medical Society, Branch of Anesthesiology, Sub-branch of Obstetric anesthesia. Consensus-based expert guidance for labor analgesia (2016). Lin Chuang Ma Zui Xue Za Zhi. 2016;32:816–8.

17. Ma YPR. Fluid management during normal delivery. Chinese Jouranl Obstet Gynecol. 2015;:316–7.

18. Ciardulli A, Saccone G, Anastasio H, Berghella V. Less-Restrictive Food Intake During Labor in Low-Risk Singleton Pregnancies. Obstet Gynecol. 2017;129:473–80.

19. Chinese Medical Society, Branch of Obstetrics and Gynecology, Sub-branch of Obstetrics. Consensus-based expert guidance for Cesarean Section (2014). Chinese Jouranl Obstet Gynecol. 2014;49:721–4.

20. Mazze R, Yogev Y, Langer O. Measuring glucose exposure and variability using continuous glucose monitoring in normal and abnormal glucose metabolism in pregnancy. J Matern Fetal Neonatal Med. 2012;25:1171–5.

21. International Association of Diabetes and Pregnancy Study Groups Consensus Panel. International Association of Diabetes and Pregnancy Study Groups Recommendations on the Diagnosis and Classification of Hyperglycemia in Pregnancy. Diabetes Care. 2010;33:676–82.

22. Cornblath M, Hawdon JM, Williams AF, Aynsley-Green A, Ward-Platt MP, Schwartz R, et al. Controversies regarding definition of neonatal hypoglycemia: Suggested operational thresholds. Pediatrics. 2000;105:1141–5.

23. Kaiser JR, Bai S, Gibson N, Holland G, Lin TM, Swearingen CJ, et al. Association between transient newborn hypoglycemia and fourth-grade achievement test proficiency: A population-based study. JAMA Pediatr. 2015;169:913–21.

24. Committee on Fetus and Newborn, Adamkin DH. Postnatal glucose homeostasis in late-preterm and term infants. Pediatrics. 2011;127:575–9.

25. Ogilvy-Stuart AL, Beardsall K. Management of hyperglycaemia in the preterm infant. Arch Dis Child Fetal Neonatal Ed. 2010;95:F126-31.

26. David E. Longnecker, David L. Brown MFN. Anesthesiology. 2ed edition. Pennsylvania, McGraw-Hill Education; 2012.

27. Harris PA, Taylor R, Thielke R, Payne J, Gonzalez N, Conde JG. Research electronic data capture (REDCap)--a metadata-driven methodology and workflow process for providing translational research informatics support. J Biomed Inform. 2009;42:377–81.

28. Austin PC. Balance diagnostics for comparing the distribution of baseline covariates between treatment groups in propensity-score matched samples. Stat Med. 2009;28:3083–107.
