# Supplementary material for: Effect of a carbohydrate-rich beverage on rate of cesarean delivery in primigravidae with epidural labor analgesia: a multicenter randomized trial
Source: BMC Pregnancy Childbirth. 2022 Apr 19;22:339. doi: 10.1186/s12884-022-04659-2 (PMC9019984; doi:10.1186/s12884-022-04659-2)
Supplement: Supplementary file 2 — Additional file 2: Table S1. Enrollment by site. [file 12884_2022_4659_MOESM2_ESM.docx]

Table S1. Enrollment by study site

| Study centers | City | Province | Number (%) |
| --- | --- | --- | --- |
| Peking University First Hospital | Beijing | Beijing | 425 (21.8%) |
| Tangshan Maternity and Child Health Care Hospital | Tangshan | Hebei | 299 (15.3%) |
| Woman's Hospital of Nanjing Medical University | Nanjing | Jiangsu | 199 (10.2%) |
| Linyi people's hospital | Linyi | Shandong | 196 (10.0%) |
| Anhui Women and Child Health Care Hospital | Hefei | Anhui | 180 (9.2%) |
| Foshan Maternal and Child Health Hospital | Foshan | Guangdong | 199 (10.2%) |
| Women's Hospital of Zhejiang University | Hangzhou | Zhejiang | 60 (3.1%) |
| Gansu Provincial Maternity and Childcare Hospital | Lanzhou | Gansu | 199 (10.2%) |
| Urumqi women and child health care hospital | Urumqi | Xinjiang | 196 (10.0%) |
| Total |  |  | 1953 (100.0%) |
